# Post-operative hearing among patients with labyrinthine fistula as a complication of cholesteatoma using “under water technique”

**DOI:** 10.1007/s00405-021-07058-z

**Published:** 2021-09-20

**Authors:** K. Thangavelu, R. Weiß, J. Mueller-Mazzotta, M. Schulze, B. A. Stuck, K. Reimann

**Affiliations:** 1 Department of Otorhinolaryngology, Head and Neck Surgery, Klinik Für HNO-Heilkunde, University Hospital Marburg, Philipps-Universität Marburg, Baldingerstrasse, 35043 Marburg, Germany; 2 Department of Neuroradiology, University Hospital Marburg, Philipps-Universität Marburg, Baldingerstrasse, 35043 Marburg, Germany

**Keywords:** Cholesteatoma, Under-water technique, Labyrinthine fistula, Post-operative hearing

## Abstract

**Introduction:**

During surgery in patients with labyrinthine fistula the mandatory complete removal of the cholesteatoma while preserving inner ear and vestibular function is a challenge. Options so far have been either the complete removal of the cholesteatoma or leaving the matrix on the fistula. We evaluated an alternative “under water” surgical technique for complete cholesteatoma resection, in terms of preservation of postoperative inner ear and vestibular function.

**Methods:**

From 2013 to 2019, 20 patients with labyrinthine fistula due to cholesteatoma were operated. We used the canal wall down approach and removal of matrix on the fistula was done as the last step during surgery using the “under water technique”. The pre and postoperative hearing tests and the vestibular function were retrospectively examined.

**Results:**

There was no significant difference between pre and post-operative bone conduction thresholds; 20% experienced an improvement of more than 10 dB, with none experiencing a postoperative worsening of sensorineural hearing loss. Among seven patients who presented with vertigo, two had transient vertigo postoperatively but eventually recovered.

**Conclusion:**

Our data show that the “under water technique” for cholesteatoma removal at the labyrinthine fistula is a viable option in the preservation of inner ear function and facilitating complete cholesteatoma removal.

## Introduction

Cholesteatoma of the middle ear is a progressive, benign epithelial lesion, characterized by an expanding growth consisting of keratinizing squamous epithelium in the middle ear and/or mastoid [1]. They are found to show intense cell proliferation accompanied by the consequent accumulation of keratin debris leading to the destruction of bony structures surrounding the temporal bone [2]. The bone eroding property of the cholesteatoma has been found to cause destruction of middle ear and inner ear structures including various intracranial and extracranial complications [3]. One of the well-known complications of untreated middle ear cholesteatoma is the erosion of the bony labyrinth resulting in a fistula of the labyrinth, which has a reported incidence of 4–12.7% [4]. A Cholesteatoma-induced inner ear fistula commonly involves the lateral semicircular canal, and to lesser extent other semicircular canals, and very rarely the cochlea itself [5]. The loss of the overlying bone structure allows pressure or mass-induced motion of the underlying endosteum, perilymph, and the endolymphatic compartment, causing vestibular and sometimes auditory symptoms [6].

Surgical removal of the cholesteatoma is the standard treatment. The specific method to be used in case of cholesteatoma with a labyrinthine fistula has been debated. Several cases with acute sensory hearing loss due to the leakage of perilymph after removal of the cholesteatoma matrix have been reported [7, 8]. Two potential surgical methods have been widely followed and debated each with its own advantages and disadvantages: (1) complete removal of the cholesteatoma with the matrix followed by repair of the bony defect in a single or two stage procedure or (2) Removal of the cholesteatoma leaving the matrix intact over the fistula (matrix preservation technique). Completely removing the matrix means a high risk of opening up the fistula, resulting in perilymph leakage and a potential irreversible sensory neural hearing loss even after patching up the fistula. On the other hand, leaving the matrix over the fistula may result in lesser disease clearance and eventually residual cholesteatoma [9, 10]. Alternatively, dissection and removal of matrix including exposure and closure of the bony defect under constant saline water irrigation has been suggested as a possible method wherein complete disease clearance can be achieved with much lesser risk of sensory hearing loss. This method of surgery named the “under water technique” was first described in 2014 and is yet to be studied in bigger cohorts [11].

Here we introduce the effectiveness of the “under water technique” in a cohort of patients (*n* = 20). We hypothesized that applying this technique facilitates complete cholesteatoma removal, while preserving the labyrinth function.

## Methods

We conducted a retrospective study in a tertiary care center in Germany. Medical records of all patients who underwent surgery for middle ear cholesteatoma from January 2013 to December 2019 were examined. We included patients with a preoperative diagnosis of labyrinthine fistula confirmed by temporal bone high-resolution Computed Tomography (CT) scan.

### Preoperative procedures

A detailed history-taking was performed, and the clinical symptoms of the patients were recorded. All patients underwent preoperative clinical ear examination, a Pure Tone Audiogram and temporal bone high-resolution CT scan. In Pure Tone Audiogram, bone conduction (BC) and air conduction (AC) thresholds at frequencies of 250, 500, 1000, 2000, 4000 and 8000 Hz were measured. In patients who presented with vertigo, vestibular function test including caloric test, electro und videonystagmography and video head impulse test was done additionally. All of the patients with a fistula were treated with intravenous corticosteroids (250 mg Prednisolone, Solu-Decortin H, Merck, Darmstadt, Germany once daily from the diagnosis of fistula up to 3 to 5 days postoperatively) and intravenous antibiotics (cefuroxime 1.5 g thrice daily from the time of diagnosis of fistula up to 5–7 days postoperatively).

### Surgical technique

**Fig. 1:**
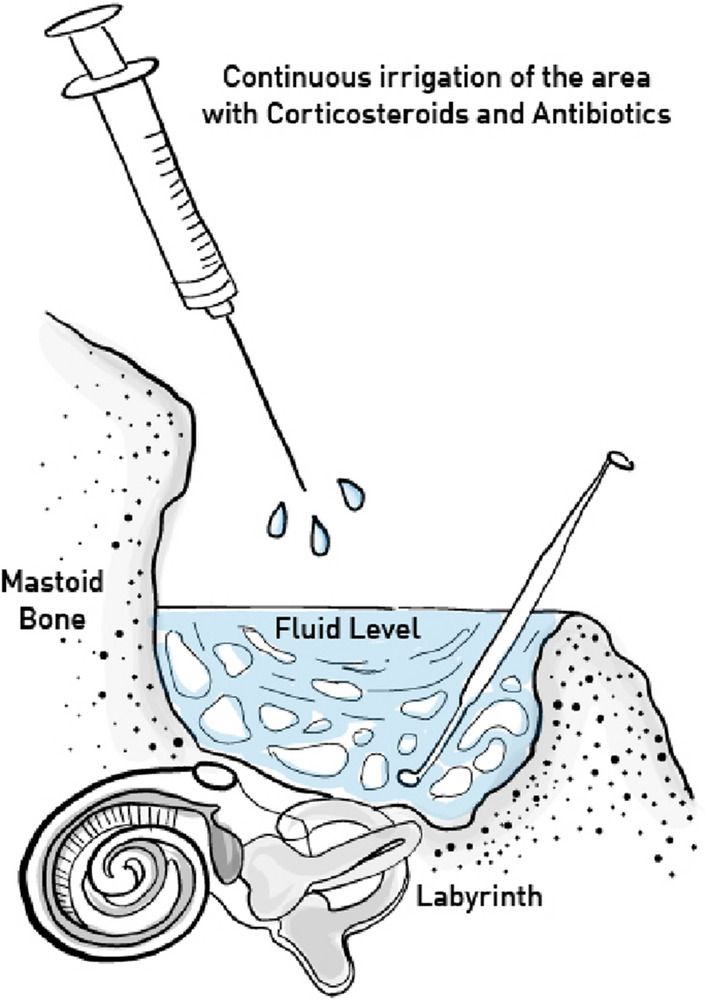
Schematic showing ‘under water’ technique

**Fig. 2:**
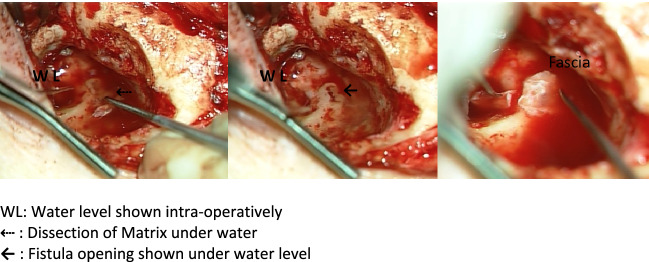
Intraoperative steps showing ‘under water’ technique as the matrix is removed and fistula repaired. *WL* Water level shown intra-operatively. **⇠** Dissection of Matrix under water. ←  Fistula opening shown under water level

All patients underwent tympanoplasty via a retro-auricular approach and a canal wall down technique for cholesteatoma removal under general anesthesia. The removal of matrix and perimatrix at the site of the fistula was done as the last operative step of the cholesteatoma removal surgery. Throughout this step, saline containing mixture of corticosteroids (prednisolone) and antibiotics (cefuroxime) was continuously superfused on to the area of surgery, creating a complete protective cover over the fistula and avoiding leakage of the perilymph, so that the removal of matrix and perimatrix was performed “under water” (Figs. 1, 2).

Fistulas were classified intraoperatively according the degree of labyrinth involvement using the Dornhoffer and Milewski classification [12]. We used temporal fascia for immediate coverage of the bony labyrinth defect. Bone pâté and fibrin-glue were used for further reconstruction. In case a ossicular replacement prosthesis was needed due to erosion of the ossicular chain, this was performed in a second surgery usually 1 year after initial cholesteatoma removal. This was to prevent a ‘third window’ at the repaired fistula.

### Post-operative procedures

The patients underwent Pure Tone Audiogram measuring BC on the first post-operative day. Further audiograms measuring both BC and AC, and vestibular function tests were then performed 6 weeks after surgery and again at various times depending on symptoms, whether or not reconstructive surgery was done.

### Statistical analysis

Mean results for audiograms and percentage hearing loss before and after surgery were compared using Wilcoxon test for pair differences. To compare the difference of percentage hearing loss before and after surgery of patients, the Mann–Whitney *U* test was applied. Group differences were considered significant if p value was less than 0.05. All analyses were performed using Stata 14.0 (StataCorp. 2014. *Stata Statistical Software: Release 14*. College Station, TX: StataCorp LP).

## Results

### Demographics

A total of 458 patients had undergone surgery for the removal of cholesteatoma between 2013 and 2019. Among them, a total 20 (4.4%) patients were pre-operatively or intra-operatively diagnosed with labyrinthine fistula (Table 1). The average age of our cohort was 45 years (± 21.1). Seven of the 20 patients were women (35%).

**Table 1:** Descriptives of the patients in our study

Variable	Classification	Number (%)
Gender	Male	13 (65)
	Female	7 (35)
Side	Right	11 (55)
	left	9 (45)
Type (Dornhoffer)*	I	5 (23)
	II	7 (32)
	III	10 (45)
Presentation	With vertigo	7 (35)
	Without vertigo	13 (65)

^*^2 Patients had fistula in two areas

**Fig. 3:**
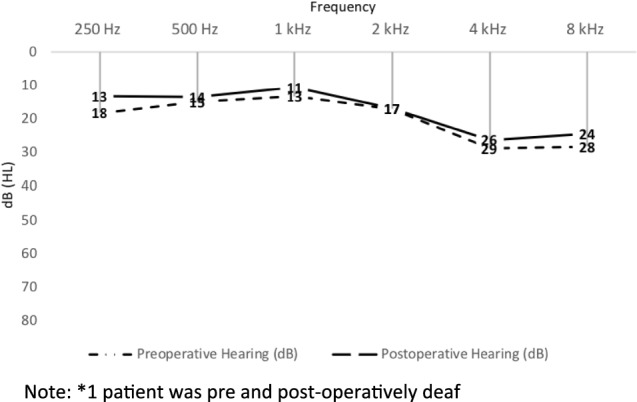
Average pre and post-operative mean bone conduction threshold in 19* patients.*1 patient was pre and post-operatively deaf

Six of the 20 patients presented with inner ear symptoms (sensorineural hearing loss and vertigo) and 1 patient presented only with vertigo (Table 1). In these seven patients the fistula was diagnosed with a pre-operative high-resolution temporal bone CT with two patients having a positive fistula test. The remaining 13 patients did not present with vertigo, but rather with symptoms of chronic otitis media (discharge from the ear and varied hearing loss) and clinically identified cholesteatoma. Fistula in these cases was diagnosed during routine pre-operative CT scan performed before surgery (Fig. 3). The cholesteatoma and the fistula were on the right side in 11 (55%) patients and on the left in the remaining 9 (45%) patients. 14 (70%) of the patients had a primary surgery, while the rest (6) were revision surgeries (Table 2).

**Table 2:** Post-operative results

Characteristics	Number (%)
Post-operative changes in bone conduction	
No change (Difference less than 10 dB)	14 (70)
Improvement more than or equal to 10 dB	4 (20)
Pre and post op deaf	2 (10)
Vertigo post-operative (*n* = 7)	2 (28)
Recurrence (*n* = 20)	4 (20)

### Intraoperative findings

All patients underwent one-stage surgery with cholesteatoma removal and closure of the fistula being performed at the same time. The same senior surgeon (R.W.) performed all surgeries. In 15 of the 20 patients, the lateral semicircular canal was involved, in two patients the superior semicircular canal, both lateral and superior semicircular canal in one patient, and both superior and footplate of stapes in one patient. Bony erosion was identified only near the stapes footplate in one patient. Fistulas were classified intra-operatively as type I (*n* = 4), type II (*n* = 5) and type III (*n* = 6), based on the Dornhoffer and Milewski. Another two patients they had fistulas in more than one place [12].

In all patients, the perimatrix and matrix were completely removed. In 13 patients the ossicle chain was removed due to erosion. In these patients, ossicle reconstruction was performed as a second step one year after the initial surgery using middle ear prosthesis. Eight of the remaining patients received hearing aid since a second surgery was declined.

### Postoperative outcome

No significant post-operative complications were noted in any of the patients. Facial paralysis was not recorded in any of the cases and there was no post-operative hearing loss. Despite the usage of intravenous corticosteroids pre-, intra- and post-operatively no associated complications such as worsening of arterial hypertension, peptic ulcer or elevated blood sugar levels were noted. The preoperative and post-operative hearing levels are shown in Fig. 3. There was no statistically significant difference between average preoperative and post-operative bone conduction thresholds across various frequencies (*p* > 0.05). 14 patients had a difference less than 10 dB. The hearing in four patients improved by more than 10 dB, while two patients who were preoperatively functionally deaf remained deaf after 6 weeks. One of these patients received cochlear implantation 6 months later. None of the patients experienced worsening of sensorineural hearing loss postoperatively.

The mean follow-up period of the patients was 45.3 months (± 25.9). Four patients developed recurrence of cholesteatoma, 3 of them were detected during the 12th postoperative month during a routine planned second look surgery, and one at the 16th postoperative month, necessitating revision surgery. None of the recurrences occurred at the site of the fistula.

**Fig. 4:**
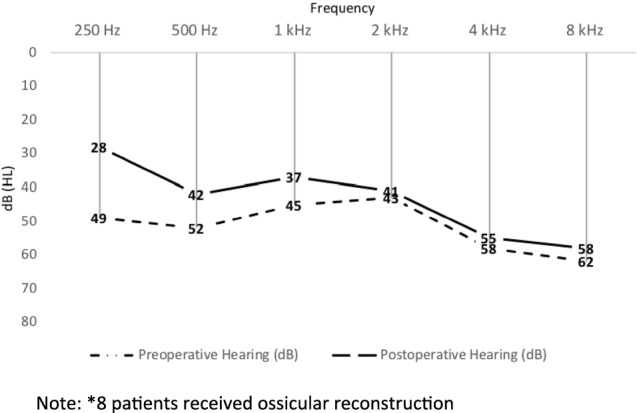
Average pre and post-operative mean air conduction threshold in 19* patients after ossiculoplasty.*8 patients received ossicular reconstruction

Although there was improvement in average air conduction threshold across lower frequencies, especially 250, 500 Hz and 1 kHz postoperatively, compared to preoperative air conduction thresholds in eight patients who received hearing rehabilitation surgery after 1 year, the difference was not statistically significant (Fig. 4).

### Pre and post-operative vestibular function

**Fig. 5:**
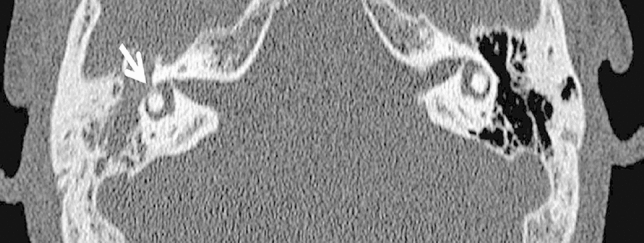
Preoperative CT scan of the temporal bone from one of the patients in the cohort showing erosion of the lateral semicircular canal

Among the seven (35%) patients who presented with vertigo preoperatively and showed pathological vestibular function tests, two (29%) patients continued to have vertigo symptoms postoperatively. A vestibular hypofunction was diagnosed in both of them postoperatively using caloric reflex test and videonystagmography and video head impulse test. One of them retained vertigo for a few weeks, with vestibular function tests normalizing in about 2 months after the surgery. The other retained vertigo for about 4 months after which vestibular function tests normalized (Fig. 5).

## Discussion

The 4.4% incidence of labyrinthine fistula in our cohort of patients with cholesteatoma is found to be on the lower end of the spectrum, when compared to the wide range of 2.7–13% reported in the literature [13, 14]. The majority of the cases (90%) reported fistula in the lateral semicircular canal reinforcing that the lateral semicircular canal is the most common site for labyrinthine fistula [15, 16].

When compared to other studies [14, 17], labyrinthine fistula in our study had a higher incidence in males (65%) than in females (35%). Similar to other studies, our results revealed no significant difference between the involvement of the right (55%) or left ear (45%) [12]. In our study, 14 (70%) patients received primary surgeries. We had a lower rate of fistula detection in revision surgeries (*n* = 6 patients, 30%), when compared to what is reported in the literature [17]. Soda-Merhy et al. performed primary and revision surgeries in 59 and 41% of patients, respectively. This study had a longer duration (144 months) and follow-up period (13 years), which may explain the difference. The follow-up period in our study was 45.3 months [17].

The majority of our patients presented with symptoms of chronic otitis media (65%) such as chronic ear pain, discharging ear and varying hearing loss. Only 35% presented with vertigo initially, raising clinical suspicion of a fistula. This is in contrast to other studies, which have reported that labyrinthine fistula usually presents with vertigo [12, 15, 17, 18]. In cases with no vertigo, pre-operative CT scan plays an important role in identifying a fistula and high-resolution CT scan was routinely performed prior to surgical treatment in all patients with cholesteatoma. High resolution CT scan had a 100% detection rate of type II and type III fistulas in our study. Other studies have reported much less detection rates of fistulas using CT scan [15, 19, 20]. The protocol applied to high resolution CT scan and the thickness of the slices used during the scan could explain the higher rate of pre-operative detection in our study. Since the fistula was diagnosed preoperatively through CT scan even in cases where a fistula was not expected, there were no surprises intraoperatively and we could plan the use of the “under water technique”, thus preventing any unexpected perilymph leakage (Fig. 2).

A positive fistula sign was pre-operatively detected in only two patients (10%), in contrast to other studies that have reported more patients with a positive fistula sign (25–55%) [12, 17, 18, 21]. It could be that cholesteatoma itself, despite causing erosion, still provides a protective cover over the erosion mitigating symptoms such as vertigo and hearing loss at the time of presentation, and patients may start showing symptoms only after its removal. Another possible explanation might be there was no control over the data recorded since our study was a retrospective collection of information from patient files and we assumed that lack of documentation implied a negative test. In many cases, there was no documentation of the fistula test, which might also explain the lower rate of positive fistula test.

In our study we preferred the Dornhoffer [12] classification over those given by Sanna [22], Palva [21] and Quaranta [14]. Palva classification (1986) classifies the fistula into 4 types based on the amount of erosion noted. Sanna classification (1988) is the simplest based only on the size of the fistula, with definitions for small (0.5–1 mm), medium (1–2 mm) and large (> 2 mm) [22]. Dornhoffer (1995) is a slightly simplified version of Palva, also based on the erosion of bony labyrinth or the membranous labyrinth [12, 21]. It is a three-point classification system emphasizing the degree of involvement of the labyrinth. In this system, a type I fistula is considered to be an erosion of the bony labyrinth with an intact endosteum. A type II fistula is a true fistula with an opened perilymphatic space. A type III fistula is an opened perilymphatic space with concomitant involvement or destruction of the underlying membranous labyrinth.

Quaranta (2008) is the latest classification and it combines the size and the position of the fistula in the inner ear such as vestibule or cochlea or semicircular canals or the stapes footplate [14]. Dornhoffer classification is widely used. This classification may correlate better with deafness; chance of deafness increases with increasing classification type [12]. The majority of the fistulas (45%) in our study were of type III in the Dornhoffer classification. There is no clear consensus as to which type is most common, with different studies reporting and using different classifications. In general, higher grade fistulas such as type II and III are found to be diagnosed and reported more often, and some authors consider only types II and III as real fistulas [13, 23, 24].

Two operative techniques namely complete removal of cholesteatoma with the matrix and matrix preservation have led to contentious discussions. Traditionally surgeons left the matrix over the fistula to preserve the existing level of hearing without opening the labyrinth, but taking the risk of leaving a potential source of bone resorption, suppurative labyrinthitis and residual disease [7, 25]. This method was modified by others wherein the matrix was removed during a second stage surgery variously at 6 or 12 months later followed by immediate repair with previously harvested autologous tissue [26]. In the recent years though total eradication of the cholesteatoma in a one-stage surgery has been favored, wanting to prevent bone resorption and residual disease [14, 27]. Sana and others advocated the usage of the size of the fistula when deciding for the type of technique namely total removal of matrix in closed technique for a fistula of less than 2 mm, and matrix left in place with a canal wall down procedure for a fistula of more than 2 mm [18, 22]. But the removal of cholesteatoma matrix regardless of the size of the fistula always carries the risk of dead ear [7].

A systematic review from 2017 showed no difference in hearing preservation between matrix removal and matrix preservation. An analysis of the individual cohort studies in the review comparing the two procedures did not show a difference in odds ratio of hearing preservation [10]. The review also shows that an overwhelming majority of surgeons prefer to remove the matrix completely, opting for disease clearance in accordance with the general rule that direct suctioning at the defect is forbidden. The go-to surgical technique explained in many studies is removal of the cholesteatoma up to the matrix, when the fistula is identified. The removal of the matrix over the fistula is done as a last step, when the matrix is gently peeled while avoiding accidental suctioning of the perilymph. The fistula is closed with bone dust, fibrin glue and/or temporal fascia.

There are very few retrospective studies comparing cholesteatoma removal while leaving the matrix intact and complete cholesteatoma matrix removal [22, 28, 29]. Most of these studies do not give details about residual disease or cholesteatoma recurrence. Meyer et al. in 2015 reported a residual rate of 19%, Geerse et al. in 2017 reported a recurrence rate of 14% across a median follow-up rate of 18 months, and although a residual cholesteatoma of 4% was reported, none of them where at the site of fistula [9, 30]. Both groups did a complete removal of the cholesteatoma matrix.

The technique of dissecting the matrix under a fluid level is rarely mentioned in published research. As far as we know, this is the only cohort to explore the effects of an alternative “under-water” technique as opposed to the two techniques often cited in the literature, demonstrating a clear success rate in preserving postoperative hearing.

Yamauchi et all in 2014 describe for the first time the under-water dissection of the matrix with endoscope using saline water as a case report [11]. Misale et al. (2019) reported a procedure called ‘hydrodissection’, where 30 patients were operated and the matrix overlying the fistula was removed under constant irrigation of saline, followed by repair with bone wax and/or autologous tissue [4]. The authors concluded that removal of the matrix under constant irrigation is sufficient to preserve cochlear and vestibular symptoms. Neither of the two papers mention residual or recurrent cholesteatoma rates, wherein our study showed a recurrence rate of 20% (*n* = 4), with none of them occurring at the site of the fistula, thus allowing to assume that we had no residual cholesteatoma.

The rationale hypothesized for operating under fluid level is to protect the inner ear from unexpected aeration unsettling the ion balance of the endolymph that may damage the inner ear function [31]. The other advantage is that operating under constant irrigation of fluids helps to prevent accidental perilymph suction and damage to the membranous labyrinth [4]. Fluid used for the “under water” technique was modified in our study by adding corticosteroids and antibiotics to the saline solution. The benefits of usage of corticosteroids locally in the ear has been extensively discussed and suggested [32, 33]. In addition, we also believe that local usage of antibiotics may help reduce post-operative infections such as labyrinthitis. Alternatively, a solution based on artificial perilymph could be a viable option for this technique. The usage of artificial perilymph mimicking the natural cochlear environment, would sustain cochlear homeostasis as suggested by Wangemann and Schacht [34].

Another age-old debate to be mentioned is canal wall down versus canal wall up technique. Although canal wall down technique has been used as a standard traditionally in case of labyrinthine fistula, canal wall up has been suggested more recently whenever possible, since the choice of the technique does not seem to influence the hearing outcome [35, 36]. Generally in case of cholesteatoma surgeries literature shows that disease recurrence rate is lower when canal wall down technique is used [37, 38]. There is not much in the literature comparing these two techniques specific to labyrinthine fistula. Meyer et al. (2016) although advocate for a closed technique, the majority of patients (80%) in their cohort were still operated using canal wall down technique. Canal wall down technique was adopted as a standard by us when closing fistulas because of well documented lower recurrence rates and since it offers easier access to the fistula and more working space especially when operating under a fluid level.

Standard use of intravenous corticosteroids in the treatment of labyrinthine fistula was adopted as early the 90 s and their beneficial effect has been to various levels of success demonstrated in the literature starting with Dornhoffer and Milewski [12]. Gocea et al. in 2012 and Albu et al. in 2013 demonstrated statistically significant improvement in hearing outcomes in their patients treated with systemic corticosteroids. Gocea et al. however had a small number of cases in the control group making meaningful comparisons difficult and Albu et al. found that the steroid treatment lost its significance in logistic analysis [23, 39]. Geese et al. in 2017 found no statistical difference in their study in direct comparison to other studies [9]. Thus in spite of being a standard treatment it is difficult to ascertain the benefits of treatment with intravenous corticosteroids and more studies are necessitated.

## Conclusion

The labyrinthine fistula from cholesteatoma does not always present with vertigo or positive fistula test as postulated till now. High resolution CT scan is an important pre-operative diagnostic tool in diagnosing labyrinthine fistula. The “under water” technique where in the matrix is removed under a fluid level is a viable alternative in preserving inner ear function to other two techniques used till now.
